# Safe and effective administration of T-VEC in a patient with heart transplantation and recurrent locally advanced melanoma

**DOI:** 10.1186/s40425-017-0250-5

**Published:** 2017-06-20

**Authors:** Gustavo Schvartsman, Kristen Perez, Jill E. Flynn, Jeffrey N. Myers, Hussein Tawbi

**Affiliations:** 1 Division of Cancer Medicine, The University of Texas MD Anderson Cancer Center, 1400 Holcombe Blvd, Unit 463, Houston, TX 77030 USA; 2 Department of Melanoma Medical Oncology, The University of Texas MD Anderson Cancer Center, 1400 Holcombe Blvd., Houston, TX 77030 USA; 3 Department of Head & Neck Surgery, The University of Texas MD Anderson Cancer Center, 1515 Holcombe Blvd., Houston, USA; 4 Department of Melanoma Medical Oncology, Department of Investigational Cancer Therapeutics, The University of Texas MD Anderson Cancer Center, 1400 Holcombe Blvd., Houston, TX 77030 USA

**Keywords:** Cancer, Melanoma, Immunotherapy, Allotransplant, Rejection, T-VEC

## Abstract

**Background:**

Immunotherapy plays a key role in the treatment of metastatic melanoma. Patients with autoimmune conditions and/or on immunosuppressive therapy due to orthotropic transplants, however, are systematically excluded from clinical trials. Talimogene laherparepvec (T-VEC) is the first oncolytic virus to be approved by the FDA for cancer therapy. To our knowledge, this is the first report of T-VEC being administered in the setting of an organ transplant recipient.

**Case presentation:**

Here we present the case of a patient with recurrent locally advanced cutaneous melanoma receiving salvage T-VEC therapy in the setting of orthotropic heart transplantation. After 5 cycles of therapy, no evidence of graft rejection has been observed to date, and the patient achieved a complete remission, and is currently off therapy.

**Conclusion:**

This case advocates for further investigation on the safety and efficacy of immunotherapeutic approaches, such as T-VEC, in solid organ transplant recipients.

## Background

Immunotherapy is the cornerstone of current treatment modalities for patients with recurrent or metastatic melanoma. Patients with a history of autoimmune disease and/or are on immunosuppressive therapy, therefore present as therapeutic challenges due to the concerns of systemic toxicity from administration of immunomodulatory treatments. In particular, solid organ transplantation recipients have a higher incidence of malignancies given their chronic immune suppression [1]. On the other hand, therapeutic options for their cancers are typically limited by the presence of comorbidities and the potential toxicities to allografts. In particular, immunotherapy looms quite dangerous given the serious consequences of graft rejection and organ failure that could be induced by non-specific stimulation of the immune system. Most early stage malignancies are addressed by initially lowering immune suppression to the minimal doses that still prevent rejection [2, 3]. However, the administration of agents that are explicitly designed to re-invigorate the T-cell response carries the clear risk of precipitating acute rejection, a lymphocytic infiltrative process, which could result in irreparable damage to the transplanted organ. Several cases have been reported of patients with kidney and liver transplants receiving checkpoint inhibitors, such as cytotoxic T-lymphocyte-associated protein 4 (CTLA-4) and programmed-death 1 (PD-1) inhibitors, with increased risk of rejection appearing to be more frequent on anti-PD-1 therapy [4–13]. One patient was reported to receive anti-PD-1 therapy in the context of heart transplantation, developing an acute rejection [14].

Talimogene laherparepvec (T-VEC, or Imlygic®, BioVex Inc., a subsidiary of Amgen Inc., based in Thousand Oaks, California) is an oncolytic virus approved by the US Food and Drug Administration (FDA) for the treatment of metastatic or unresectable melanoma with injectable skin or nodal lesions [15]. T-VEC is expected to induce a systemic immune response and abscopal effects have been noted with it. How robust is this immune response, and how it may affect solid organ transplant recipients receiving immunosuppressive therapy, however, is unknown. Here, we describe the first case of the safe and effective administration of T-VEC to a patient with recurrent cutaneous melanoma not eligible for PD-1 inhibitors due to a history of heart transplantation.

## Case presentation

This is a 71-year-old male with a history of orthotropic heart transplantation in 2002 due to severe coronary disease and heart failure. Until 2016, he was regularly followed by his cardiologist twice a year, with normal yearly heart catheterization and echocardiogram. His immunosuppression was achieved with cyclosporine, at 100 mg PO twice daily, and prednisone, at 5 mg PO daily. Additionally, this patient suffered from hypertension, hypercholesterolemia, insulin-dependent type 2 diabetes mellitus, depression, and had a prior ischemic stroke in 1999, with no sequelae.

**Fig. 1:**
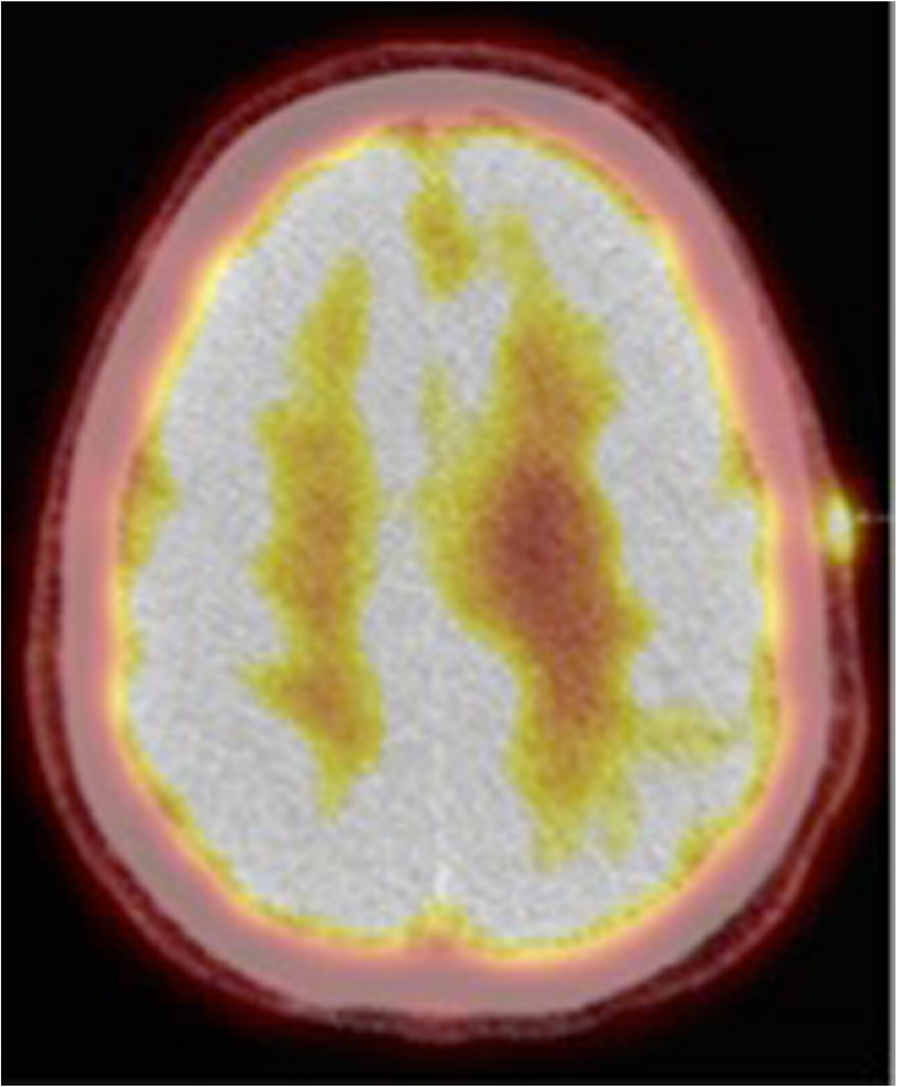
PET-CT demonstrating local recurrence in the left scalp

Since his immunosuppression started in 2002, the patient had multiple scalp and arm basal cell and squamous cell carcinomas of the skin resected. A new left scalp lesion appeared in 2015, with a biopsy demonstrating melanoma, spindle-cell type, with desmoplastic features. He underwent a wide local excision (WLE) in August/2015 at an outside facility, which contained basal cell carcinoma present at the deep margin, requiring a re-resection to achieve negative margins. Final Breslow thickness was of 3.25 mm. Tumor was incompletely staged at the time, with no sentinel lymph node biopsy performed. Shortly afterwards, in January/2016, the patient presented with a local recurrence and underwent a WLE, outer table craniectomy, left parotidectomy, and left cervical lymph node dissection. Pathology demonstrated a 10.1 mm-thick melanoma, with cancer present at the tissue margins, extensive perineural invasion, microscopic satellitosis and 0 out of 40 lymph nodes positive. A re-resection successfully obtained negative margins. At that point, patient self-referred to MD Anderson and was seen for the first time in March/2016 by our surgical team. Complete staging was obtained with a PET-CT and a brain MRI, with no visible distant disease except for a dermal in-transit metastasis inferior to the left vertex scalp resection operative site (Fig. 1). We performed molecular profiling with a 50-gene somatic mutation panel on outside tissue using a next generation sequencing platform, identifying mutations in the FGFR1, TP53 and VHL genes. BRAF, NRAS and KIT were found to be wild-type. Patient was subjected to additional resection of dermal metastases with immediate reconstruction using a free anterolateral thigh flap and a pedicled vastus lateralis muscle flap in May/2016. His cyclosporine dose was reduced to 50 mg twice daily as a recommendation by the Melanoma Medical Oncology team, as clinically tolerated for his transplant, prior to surgery. Cyclosporine was held after the operation due to post-operative acute kidney injury. Echocardiogram showed no heart abnormalities and preserved ejection fraction. After a prolonged 20-day inpatient recovery, patient was discharged on 50 mg twice daily of cyclosporine. Less than a month after surgery, however, patient developed a recurrence in the left eyebrow and left temple, biopsy-proven as the same histology as his original tumor. Patient underwent additional resections and adjuvant radiation therapy in July/16, consisting of 30 Gray in 5 fractions to the left scalp. Adjuvant immunotherapy was contraindicated due to his heart transplantation.

**Fig. 2:**
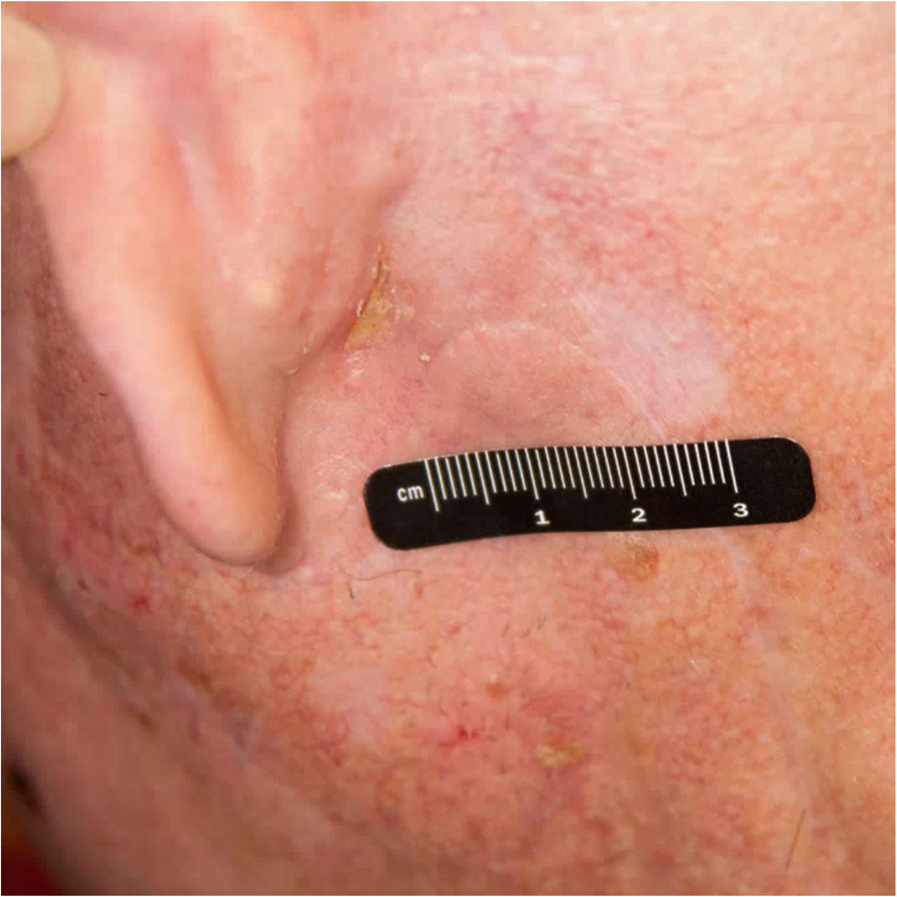
Near-resolution of left mastoid lesion. Pre-treatment image documentation was not obtained

In September/2016, unfortunately, the patient’s spindle cell melanoma recurred as a left post-auricular skin lesion. It was resected on 9/2/16, but a mastoid lesion was then visualized and biopsy once again demonstrated melanoma. After discussing risks and benefits, patient received his first dose of T-VEC on 9/14/16, consisting of a 2 ml-injection into the left mastoid tumor. The second dose was on 10/5/16. Prior to his third cycle, the patient had a clinical response in the mastoid tumor, but unfortunately developed a new palpable and tender left supraclavicular node. Fine-needle aspiration confirmed metastatic melanoma. Patient then received cycles 3 and 4 of T-VEC into the mastoid tumor and cycles 1 and 2 of therapy into the left supraclavicular node, consisting of 1 ml to each site, on 10/19/16 and 11/2/16. Upon reevaluation, he developed a significant response to both sites, with complete regression of the supraclavicular lymph node and very good partial response of the mastoid lesion (Fig. 2). However, the patient experienced progressive toxicity following every injection. After having no adverse events following the first injection, he was briefly hospitalized after the second cycle due to fatigue and fever, with slightly elevated white blood cell count (WBC). No antibiotics were administered and it self-resolved. Patient was subsequently admitted during his third and fourth injections for close monitoring, and continued to develop significant fatigue and fever that would last for around 3 days after each dose, with WBC capping at 12 K/uL. After the fourth cycle, patient presented with severe weakness, shortness of breath and fever. Pneumonia was diagnosed and patient received IV antibiotics, as well as an extra 2-week break from therapy, with good recovery. In the interim, he developed a new left anterior chest dermal deposit and, for his 5^th^ cycle of T-VEC on 11/30/16, 0.1 ml was injected into the chest and 0.9 ml into the left mastoid lesion. The latter completely regressed shortly afterwards. The patient’s chest lesion was then resected on 12/13/16 and found to be a basal cell carcinoma, rather than melanoma. Patient had a significantly faster clinical recovery from his last cycle of T-VEC. Most recent PET-CT on 03/01/2017 showed no evidence of disease and patient was discontinued from therapy due to lack of injectable lesions. During the entire course of his therapy, patient’s echocardiogram performed every 3 months showed no abnormalities whatsoever. Last follow-up visit in March, 2017 did not evidence any signs of disease.

## Discussion

Clinical trials of the efficacy of checkpoint inhibitors and T-VEC excluded patients with active autoimmune diseases or on immunosuppressive therapy due to organ transplantation [15–19]. To our knowledge, this is the first reported case of T-VEC being administered to a solid organ transplant recipient. In addition, this is the first case of a patient with a heart transplant being treated with an immune-based therapy.

Several cases were reported of the administration of agents such as ipilimumab and nivolumab in liver or kidney transplant recipients [4–13]. No patients receiving ipilimumab developed graft rejection, whereas some of the patients receiving PD1 inhibitors presented with kidney and heart rejections and even graft loss [4, 6, 10, 14], indicating that these agents should be used with more caution in patients with vital organ transplantations (liver, heart).

T-VEC is an FDA approved, first-in-class oncolytic virus based on a modified herpes simplex virus type 1 designed to selectively replicate in and lyse tumor cells while promoting regional and systemic antitumor immunity [15]. An overall response rate of 26% was observed, being 10% of those complete remissions. Patients with skin and nodal disease only were found to have improved responses and survival outcomes. The majority of responses were durable and median time to response was 4.2 months. T-VEC’s systemic immune response was demonstrated with regression of more than 50% of visceral lesions (non-injected), systemic immune-related adverse events such as vitiligo [20], and increased numbers of MART-1–specific T cells observed in metastases undergoing regression after T-VEC therapy [21]. It has also been shown to decrease CD4 + FoxP3+ regulatory T cells and CD8 + FoxP3+ suppressor T cells, finding consistent with a systemic immune response. Nonetheless, its effects are clearly superior in loco-regional disease - not only due to higher activity in injected lesions by the additional viral oncolytic effect, but also as a consequence of activation of T cells that may preferentially traffic to metastases in similar anatomic sites as those injected (skin, subcutaneous tissue, lymph nodes) [22]. One could postulate, therefore, that there might be an immune response window between levels of systemic response achieved by T-VEC and the levels required to induce a transplant rejection.

The patient above reported had a recurrent melanoma of the head and neck region, being subject to several invasive resections, large reconstructions and radiation therapy in attempt to render him disease-free, while sparing him from immunotherapy due to his heart transplant. Decreasing the intensity of his immunosuppression as tolerated, as recommended by his transplant care team [2, 3], was ineffective as well. After yet another recurrence in the left mastoid region, possible risks and benefits of T-VEC were discussed with patient and he agreed to be challenged with it. After 2 cycles, despite achieving a response in the injection site, the patient developed a new left supraclavicular node, biopsy-proven to be metastatic disease. Pseudo-progression is a phenomena frequently observed with this agent, occurring in more than 50% of cases [15]. Acknowledging this, the node also became subject to injections and, after 5 cycles, the patient achieved a complete response in both sites and was discontinued from therapy (a new chest lesion was found and also target of one injection, but was later found to be a basal cell carcinoma after excision). The patient did experience toxicity to T-VEC therapy following every cycle, requiring interruption of treatment for 2 weeks after the fourth cycle due to overlapping pneumonia. Such toxicity, however, was not unexpected, as about half the patients treated with this agent experienced flu-like symptoms (fatigue/malaise, fevers, chills) [15]. Symptoms are usually worse during the initial 3 months of therapy and tend to resolve within 72 h of onset. Toxicity leading to discontinuation of treatment occurred in only 4% of cases. His heart function was closely monitored with serial echocardiograms, with no abnormalities seen during the entirety of treatment.

## Conclusions

In conclusion, this report advocates for further investigation of the safety and efficacy of T-VEC in the setting of organ transplantation. As a preliminary finding, it seems to maintain its efficacy and be potentially safe even in patients with vital organ transplants. After failure of surgical and radiation options and without a targetable BRAF mutation, T-VEC is an option that should be discussed with patient with injectable lesions, particularly in locally advanced patients. As for our patient, he still has a high probability of relapse and will be monitored closely.

## Abbreviations

CTLA: 4 - Cytotoxic T-lymphocyte-associated protein 4
FDA: Food and Drug Administration
PD-1: Programmed-death-1
TVEC: Talimogene laherparepvec
WLE: Wide local excision
